# A Case of Rheumatoid Arthritis Presenting with Renal Thrombotic Microangiopathy Probably due to a Combination of Chronic Tacrolimus Arteriolopathy and Severe Hypertension

**DOI:** 10.1155/2019/3923190

**Published:** 2019-03-06

**Authors:** Fumika Honma, Yoshihide Fujigaki, Yoshikazu Nemoto, Hirotoshi Kikuchi, Michito Nagura, Shigeyuki Arai, Kenichi Ishizawa, Osamu Yamazaki, Yoshifuru Tamura, Fukuo Kondo, Ryuji Ohashi, Shunya Uchida, Shigeru Shibata

**Affiliations:** ^1^Department of Internal Medicine, Teikyo University School of Medicine, Itabashi-ku, Tokyo, Japan; ^2^Department of Pathology, Teikyo University Hospital, Itabashi-ku, Tokyo, Japan; ^3^Department of Diagnostic Pathology, Nippon Medical School Musashikosugi Hospital, Kawasaki, Kanagawa, Japan

## Abstract

A 51-year-old woman with rheumatoid arthritis presented with mild hypertension 20 months after tacrolimus treatment and developing proteinuria 24 months after the treatment. Tacrolimus was discontinued 27 months after the treatment, followed by heavy proteinuria, accelerated hypertension, and deteriorating renal function without ocular fundus lesions as a clinical sign of malignant hypertension. Renal biopsy revealed malignant nephrosclerosis characterized by subacute and chronic thrombotic microangiopathy (TMA), involving small arteries, arterioles, and glomeruli. Focal segmental glomerulosclerosis, probably secondary to chronic TMA, was identified as a cause of heavy proteinuria. The zonal tubulointerstitial injury caused by subacute TMA may have mainly contributed to deteriorating renal function. The presence of nodular hyalinosis in arteriolar walls was indicative of tacrolimus-associated nephrotoxicity. Together with other antihypertensive drugs, administration of aliskiren stabilized renal function with reducing proteinuria. Owing to the preexisting proteinuria prior to severe hypertension and the complex renal histopathology, we postulated that chronic TMA, which was initially triggered by tacrolimus, was aggravated by severe hypertension, resulting in overt renal TMA.

## 1. Introduction

Thrombotic microangiopathy (TMA) is a pathologic term where vascular and glomerular lesions due to endothelial damage and vascular occlusion can be observed and is characterized by a clinical presentation with thrombocytopenia, hemolytic anemia, and organ injuries, including acute kidney injury (AKI) [1]. However, localized renal TMA without systemic manifestation of TMA exists and can be diagnosed only by renal biopsy.

Severe hypertension can induce TMA within the renal vasculature typically associated with fibrinoid necrosis of arterioles and the glomerular capillary tufts [2]. The exact mechanism remains to be established, but TMA may occur when vascular autoregulation cannot accommodate the severe hypertension-induced shear stress. Severe hypertension-induced TMA showed a low incidence of thrombocytopenia and hemolytic anemia [2]. Renal function may improve or stabilize in about 50 to 80% of patients of severe or malignant hypertension with or without biopsy-proven TMA upon adequate blood pressure (BP) control [2, 3].

Calcineurin inhibitor (cyclosporine and tacrolimus)-associated TMA is a rare but well documented cause of AKI [4, 5]. Calcineurin inhibitor-associated TMA is attributed to the endothelial injury secondary to vasoconstriction, which induces ischemia, increases platelet aggregation, and activates prothrombotic factors [6]. Calcineurin inhibitor-associated TMA may often localize to the renal graft in posttransplant patients and show AKI or delayed graft function with few or no systemic manifestations of TMA [6]. Discontinuation or reduced dose of calcineurin inhibitor is the main treatment of calcineurin inhibitor-associated TMA [7].

Severe hypertension may be either a cause of TMA or a manifestation of renal involvement from an underlying TMA. About 20-40% of patients with severe/malignant hypertension presented with TMA and/or microangiopathic hemolysis [3, 8]. Thus, concomitant renal TMA and severe hypertension could raise the differential diagnosis of TMA and lead to a vicious cycle.

Here, we describe a patient of rheumatoid arthritis (RA) with a most recent history of long-term tacrolimus use, who presented with localized renal TMA in association with clinical feature of developing heavy proteinuria and severe hypertension, subsequently deteriorating renal function. We assumed that renal TMA in our case may be caused by a combination of chronic tacrolimus arteriolopathy and subsequent severe hypertension.

## 2. Case Report

**Figure 1:**
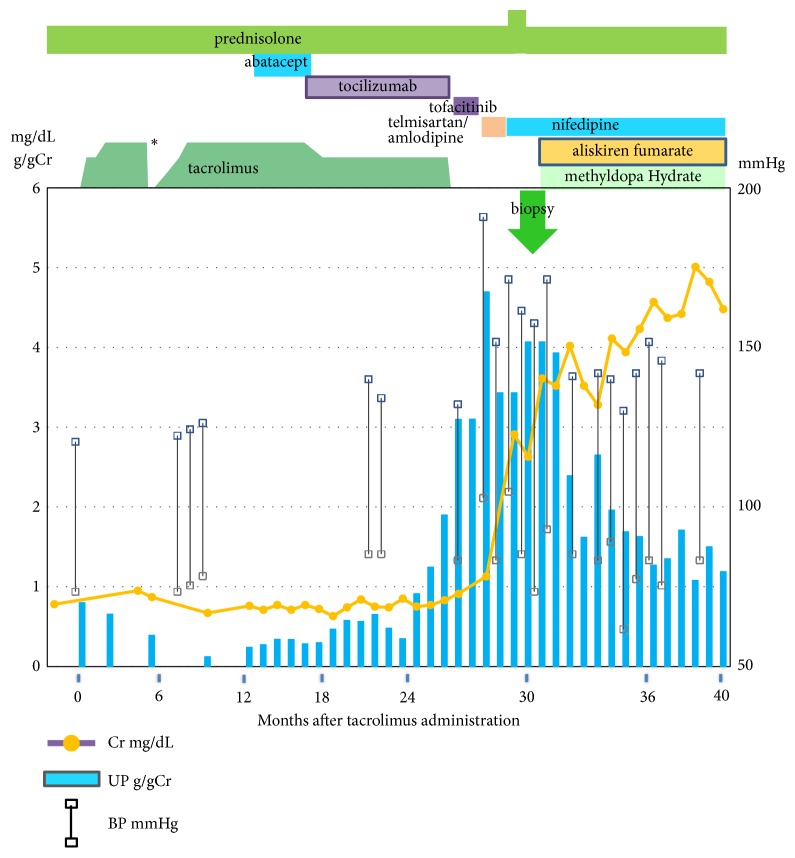
Clinical course of the patient after introduction of tacrolimus treatment. *∗*: surgical operation, Cr; creatinine, UP; proteinuria, and BP; blood pressure.

A 51-year-old Japanese woman was admitted to our hospital for the evaluation of heavy proteinuria, deteriorating renal function, and severe hypertension. She had a medical history of RA at the age of 42 and left vitrectomy for retinal detachment and bilateral femoral head replacement following fracture at the age of 49. Since she had drug allergies to many drugs, various treatments for RA were tried to introduce including methotrexate, infliximab, etanercept, salazosulfapyridine, leflunomide, bucillamine, tacrolimus, abatacept, and/or tocilizumab in addition to prednisolone (PSL) and nonsteroidal anti-inflammatory drugs. She was treated with the dosage of 2 to 3 mg/day of tacrolimus, standard dose for RA in addition to PSL 8 mg/day from the age of 48 for 2 years and 3 months. Clinical course after introduction of tacrolimus is shown in Figure 1. BP was increased from 120/70 mmHg to 140/80 mmHg 20 months after tacrolimus treatment, trough levels of tacrolimus fell within acceptable ranges between 5 and 10 ng/dL during the course. Proteinuria began to increase from the baseline proteinuria of 0.3 to 0.5 g/g creatinine 24 months after tacrolimus treatment, but serum creatinine level was sustained around 0.8 mg/dL. Tacrolimus and tocilizumab were changed to tofacitinib citrate 27 months after tacrolimus treatment because of uncontrolled arthritis of RA. However, tofacitinib citrate was discontinued 2 months after the treatment because of allergic reaction. Proteinuria was further increased after discontinuation of tacrolimus and tocilizumab, and then severe hypertension 190/100 mmHg and progressive renal dysfunction developed. 40 mg telmisartan/5 mg amlodipine besilate combination tablet was introduced 2 months after tacrolimus discontinuation. Her renal function was further deteriorated to creatinine of 2.63 mg/dL; thus she was admitted to our hospital 3 months after tacrolimus discontinuation.

On admission, body temperature was 36.5°C, height 154.0 cm, weight 44.9 kg, BP 170/102 mmHg, and pulse rate 88/min. Physical examination showed numbness in hands, pain in the elbows, wrists, knees, and metacarpophalangeal (MP) joint of the right thumb finger, swelling of MP joint in the right second finger, and mild pitting edema in bilateral legs but no abdominal bruit. She had no focus of infection and sclerotic skin lesion and no experience of Raynaud's phenomenon. The laboratory data on admission are shown in Table 1. Urinary examination showed heavy proteinuria and microscopic hematuria. Urinary low-molecular-weight proteins and urinary N-acetyl-*β*-D-glucosaminidase were elevated. Blood examination showed anemia, hypoalbuminemia, renal dysfunction, and hypocalcemia. Immunological examination indicated normocomplementemia, normal tests for anti-DNA antibody, anticardiolipin antibody, and myeloperoxidase and proteinase 3-anti-neutrophil cytoplasmic antibodies, but positive tests for RA-associated factors including rheumatoid factor, matrix metalloproteinase-3, and anti-SS-A antibody. Repeated peripheral smears showed no evidence of hemolysis. Serum renin activity and aldosterone concentration were of high value. Her hypocalcemia could be explained by use of denosumab for the treatment of steroid-induced osteoporosis.

The electrocardiogram showed voltage criteria of left ventricular hypertrophy. Chest X-ray showed no apparent cardiomegaly and lung edema. Abdominal ultrasound detected normal shape and size in the kidneys and multiple hemangioma in the liver. Echocardiography revealed ejection fraction 56% Simpson method, ratio of E to e' 22.6, and left ventricular wall thickening. Fundoscopy did not show exudate hemorrhage and papilledema.

**Figure 2:**
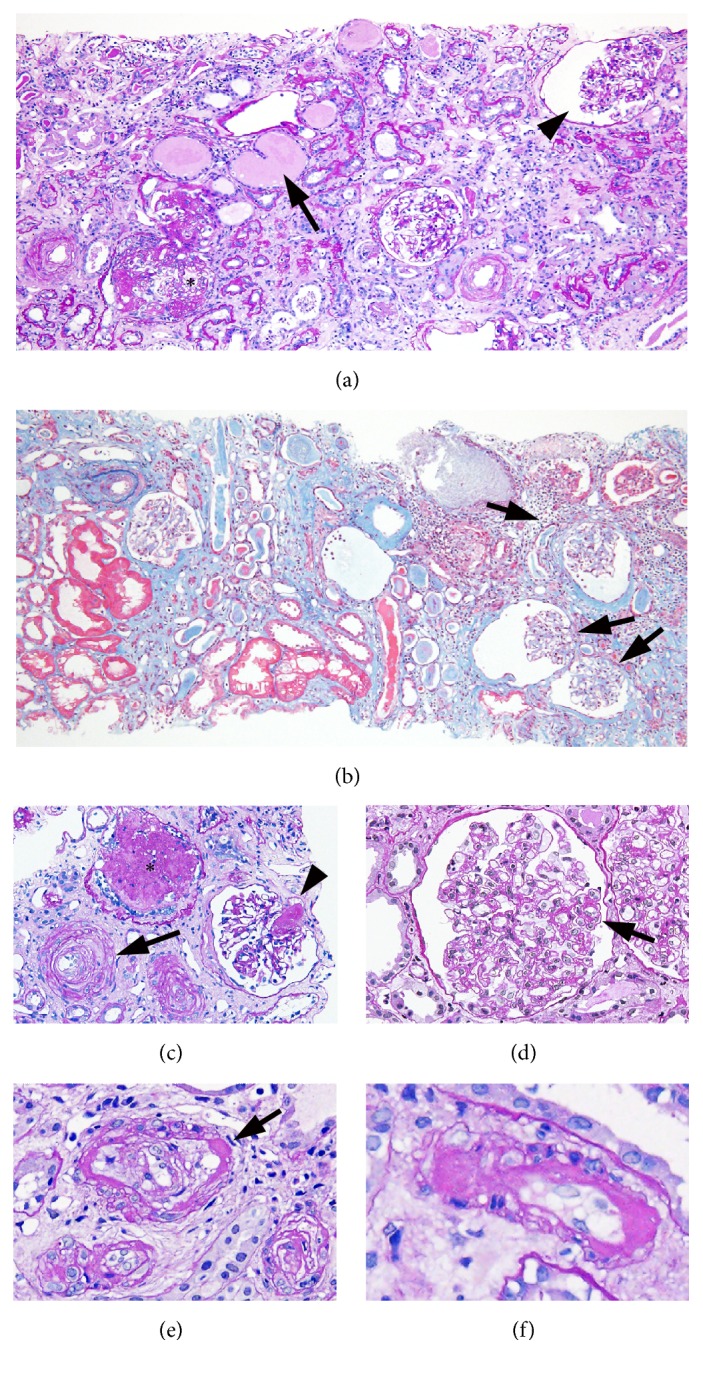
Light micrographs of renal tissues. (a) A glomerulus (asterisk) is mildly enlarged, showing focal segmental glomerulosclerosis while another glomerulus exhibits collapsed capillary tuft (arrowhead). Dilated tubules with flattened epithelial cells containing proteinaceous casts are present (arrow) (periodic acid-Schiff stain; original magnification x200). (b) Several glomeruli have collapsed capillary tufts (arrows). There is an extensive striped pattern of interstitial fibrosis and tubular atrophy (Elastica–Masson staining; original magnification x200). (c) A glomerulus is globally sclerotic (asterisk). There is organized thrombus formation within an afferent arteriolar lumen (arrowhead). Concentric intimal hyperplasia of arterioles forming “onion skin” lesion is noted (arrow) (periodic acid-Schiff stain; original magnification x200). (d) Glomerular capillary wall is segmentally thickened showing double contour (arrow) (periodic acid-Schiff stain; original magnification x400). (e) Arteriolar lumina are occluded by fibrous intimal thickening with recanalization. Nodular hyalinosis of arterial walls is noted (arrow) (periodic acid-Schiff stain; original magnification x400). (f) An arteriolar wall shows circumferential and transmural hyalinosis forming peripheral nodules (periodic acid-Schiff stain; original magnification x400).

**Figure 3:**
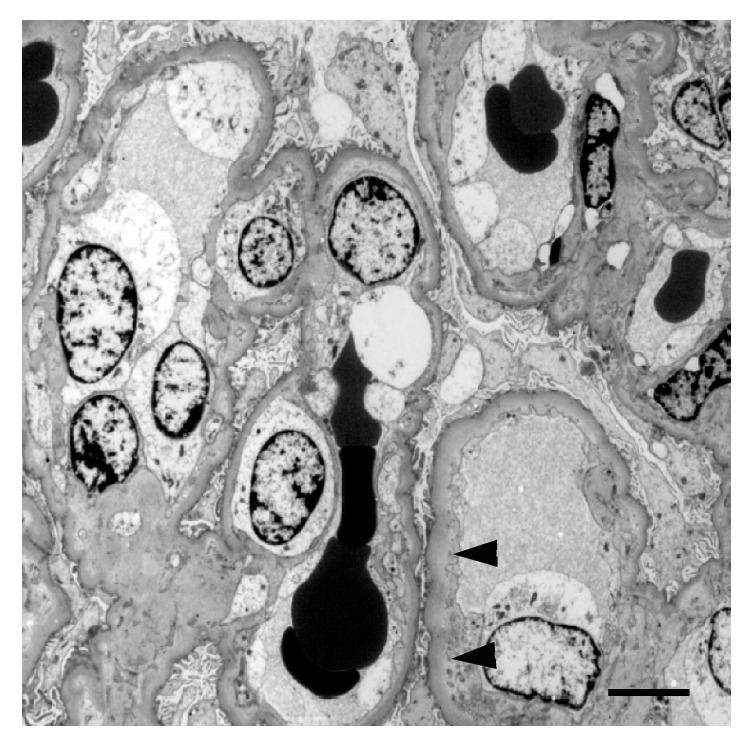
Electron micrograph of a glomerulus. Swollen glomerular endothelial cells with loss of fenestrations, irregularity thickened lamina rara interna with small remnants in the subendothelial spaces (arrowheads), and partial foot process effacement are seen. Bar=5.0 *μ*m.

With a clinical suspicion of secondary amyloidosis, focal segmental glomerulosclerosis (FSGS), or malignant nephrosclerosis, renal biopsy was performed. A renal biopsy showed 5 glomeruli with adhesive lesions and segmental sclerosis or global sclerosis and 8 glomeruli with ischemic shrinkage of glomerular tufts out of 22 obtained glomeruli (Figures 2(a), 2(b), and 2(c)). Some of the remaining glomeruli showed collapse of capillary tufts (Figures 2(a) and 2(b)), FSGS (Figure 2(a)), and segmental thickening of capillary walls showing double contour (Figure 2(d)). There were extensive tubular atrophy and interstitial edema to fibrosis involving 70% of renal parenchyma, accompanied by chronic and acute inflammatory cell infiltration (Figures 2(a) and 2(b)). Distribution of the tubulointerstitial damage was zonal, indicative of ischemic injury following vascular compromise (Figure 2(b)). The afferent arteriole of the glomerulus was occluded by an organized thrombus, and the arterioles showed concentric intimal hyperplasia forming “onion skin” lesion (Figure 2(c)). Some of the small arterial and arteriolar lumina were markedly narrowed by thickened fibrous intima (Figure 2(e)). Of note, some arteriolar walls exhibited circumferential and transmural nodular hyalinosis (Figures 2(e) and 2(f)). An immunofluorescence study showed nonspecific segmental staining of IgM, C1q, and C3 in glomeruli, and IgA and IgM in tubular casts. Electron microscopy revealed swollen glomerular endothelial cells with loss of fenestrations, irregularly thickened lamina rara interna, and foot process effacement involving 30% of podocytes (Figure 3). No electron dense deposit was identified. Collectively, these histological findings are suggestive of malignant nephrosclerosis and tubulointerstitial damage, represented by subacute/chronic TMA.

Severe hypertension and tacrolimus use were considered to be causes of TMA in our patient. Since tacrolimus had already been withdrawn, we tried to manage blood pressure on an appropriate level. It is reported that hypertension is highly prevalent among patients with RA, and use of anti-inflammatory analgesics and disease-modifying drugs with hypertensive potential, and yet to be determined inflammatory pathways, and genetic factors may synergistically lead to hypertension [9]. Nonsteroidal anti-inflammatory drugs and tofacitinib citrate [10] might have contributed to severe hypertension in our patient. However, it is more likely that her severe hypertension may have been caused by renal parenchymal damage with marked activation of renin-angiotensin-aldosterone system. To control severe hypertension, amlodipine besilate was changed to nifedipine. In addition, methyldopa and also aliskiren to inhibit renin-angiotensin system were introduced, then BP was gradually decreased. After aliskiren was administered, renin activity was reduced from 12 to 0.6 ng/mL/h and aldosterone concentration from 242 to 69.4 pg/mL in one week. Blood pressure and renal function eventually stabilized with gradual reduction of proteinuria. One year after renal biopsy, serum creatinine was 4.03 mg/dL and proteinuria was 1.0 g/g creatinine (Figure 1).

## 3. Discussion

Causes of TMA including hemolytic uremic syndrome, thrombotic thrombocytopenic purpura, and atypical hemolytic uremic syndrome, which were almost uniformly include thrombocytopenia and hemolytic anemia, were excluded in our patient. Thus, we did not examine ADAMTS13 (a disintegrin and metalloprotease with thrombospondin-1-like domains) activity. As for secondary TMA, she had no clinical evidence of infection. She had no skin lesion, and anti-SCL-70 and anti-centromere antibodies were negative 2 and a half years ago. This may support the fact that scleroderma renal crisis is excluded. Our patient had conditions including RA, tacrolimus use, and severe hypertension which could raise TMA. However, we postulated that renal TMA in our patient was not caused by severe hypertension alone but by a combination of chronic tacrolimus arteriolopathy and severe hypertension. This could be explained as follows.

First, our patient had severe hypertension, but the absence of fundus lesion, which did not fulfill the diagnosis of accelerated/malignant hypertension. Renal histology of accelerated/malignant hypertension shows TMA involving intrarenal fibrinoid necrosis of arterioles, containing the necrotic remnants of the smooth muscle cells as well as fibrin and platelet deposits [11]. Another type of lesion is a proliferative endarteritis of medium and small sized arteries and arterioles, containing proliferated smooth muscle cells and myofibroblasts. This lesion is called “onion skin” [11]. Though hyaline thrombus in one arteriole and onion skin in arterioles were observed in our patient, fibrinoid necrosis of renal arterioles, which is often observed in the acute phase of malignant hypertension-associated TMA, was not found. Signs of nephritis due to severe renal ischemia usually appear in parallel with severe hypertension in patients with malignant hypertension [11]. Unlike this clinical manifestation, our patient showed the development of proteinuria before severe hypertension occurred. These facts suggest that renal TMA was not merely caused by severe hypertension, but also by other exacerbating factors in the current case.

Second, the renal biopsy showed features of chronic TMA, characterized by double contours of glomerular capillary walls, and fibrous thickening of arterial walls with organized or recanalized thrombi, and nodular hyalinosis of arteriolar walls. Given the clinical course of the patient, the presence of these chronic changes cannot be explained by short-term severe hypertension alone. As these chronic lesions can reportedly be induced by calcineurin-associated nephrotoxicity [6, 12], it is probable that the chronic lesions of our case may have appeared as part of tacrolimus-associated TMA. It is reported that chronic calcineurin inhibitor nephrotoxicity is clinically characterized by hypertension, progressive renal insufficiency, and variable degree of proteinuria. Histologically, hyaline arteriolopathy, striped interstitial fibrosis with proportional tubular atrophy, and glomerulosclerosis are often found [12] like the present case. In calcineurin inhibitor-associated TMA among postrenal transplant patients, it is emphasized that TMA is confined to the kidney with little or no systemic evidence of the thromboangiopathic process, underscoring the importance of the graft biopsy in making the diagnosis [6]. Secondary FSGS in our patient could be caused by chronic calcineurin inhibitor nephrotoxicity and/or severe/malignant hypertension [13, 14]. It is noteworthy that normal donor kidneys of kidney-pancreas transplant recipients during tacrolimus based therapy with tacrolimus concentration averaged 7.9 ± 3.9 ng/dL showed chronic arteriolar toxicity in 4.3%, 33.6%, and 77.2% after 1-, 5-, and 10-year treatment, respectively [15]. The authors suggested that cumulative nephrotoxicity from prolonged tacrolimus exposure over years-to-decades remains the best explanation in their cohort. Earlier increasing proteinuria, raising BP, and subsequently deteriorating renal function may rather suspect that TMA lesions were made possibly by a contribution of chronic, smoldering tacrolimus arteriolopathy [16] and severe hypertension. Though nodular hyalinosis is not specific for calcineurin inhibitor nephrotoxicity, it is conceivable that nodular hyalinosis in our patient may indicate chronic tacrolimus nephrotoxicity [17].

Lastly, since our patient suffered from RA, secondary TMA associated with RA should be considered. Except for rheumatoid vasculitis only a few cases with RA were reported to be associated with systemic TMA not localized renal TMA [18, 19], all which were thought to be caused by severe deficiency of ADAMTS13 due to autoantibodies that alter its function [20]. Since there were no renal vasculitic lesions, RA-associated TMA was excluded in our patient.

Taken together, since glomerular and tubulointerstitial lesions in our patient were too severe and chronic for her recorded BP to explain the lesions, we postulated that long-term tacrolimus arteriolopathy with smoldering renal TMA caused at least in part secondary FSGS with heavy proteinuria and hypertension [13]. Subsequently, even after discontinuation of tacrolimus, severe hypertension, not malignant hypertension level due to developing renal parenchymal damage further damaged arteriolar endothelium, resulting in overt renal TMA with deteriorating renal function. The zonal tubulointerstitial injury caused by subacute TMA may have mainly contributed to deteriorating renal function. Interestingly JAK3-STAT pathway has been claimed to be a responsible pathway in some forms of glomerulonephritis. Targeting of this pathway has been reported to be effective in the treatment of experimental lupus nephritis [21]. On the other hand disturbances of renal function have been reported with use of tofacitinib, a first-generation JAK inhibitor [22]. Therefore, increase in serum creatinine was also concerned in our patient with RA using tofacitinib; however, recent clinical trial reported that slightly decrease in measured glomerular filtration rate 6 weeks after tofacitinib treatment retuned towards baseline after tofacitinib discontinuation with no significant difference versus placebo [23]. Accordingly, it is conceivable that BP control with renin-angiotensin system inhibition could not ameliorate renal dysfunction due to the preexistence of chronic renal damage but stabilize renal function with reducing proteinuria in our patient.

In summary, some subsets of patients who receive tacrolimus for a long time may be suffered from heavy proteinuria, severe hypertension associated with progressive deterioration of renal function due to renal TMA even after withdrawal of tacrolimus. Severe hypertension must act as a subsequent aggressor to intensify damage of endothelial cells in small arteries, arterioles, and glomeruli, which had been already injured by tacrolimus arteriolopathy, leading to overt renal TMA. Physician should keep in mind that tacrolimus can insidiously evoke chronic smoldering renal TMA.

## Figures and Tables

**Table 1:** Laboratory data on admission.

*Urinalysis *
Gravity 1.014
pH 6.0
Protein 3+
Glucose (-)
Occult blood trace
*Sediments *
Red blood cell 1-4 /high power field
Wight blood cell 10-19 /high power field
*Urine chemistry *
Protein 245 mg/dL
Creatinine 60.2 mg/dL
N-acetyl-*β*-D-glucosaminidase 12.5 U/L
*β*2-microglobulin 40,066 *μ*g/L
*Complete blood count *
WBC 15,000 /*μ*L
Hb 10.6 g/dL
Platelet 36.8×10^4^/*μ*L
*Blood chemistry *
Albumin 2.7 g/dL
Lactate dehydrogenase 385 IU/L
Urea nitrogen 46.9 mg/dL
Creatinine 2.86 mg/dL
Uric acid 6.9 mg/dL
Na 139 mEq/L
K 3.4 mEq/L
Cl 103 mEq/L
Ca 6.8 mg/dL
Pi 1.8 mg/dL
Estimated GFR 14.7 ml/min/1.73m^2^
*Serology *
HBs antigen 0.1 C.O.I
HCV antibody 0.1 C.O.I
IgG 621 mg/dL
IgA 251 mg/dL
IgM 152 mg/dL
CH50 60< U/mL
C3 110 mg/dL
C4 30 mg/dL
C-reactive protein 2.87 mg/dL
Rheumatoid factor 24.2 U/mL
MMP-3 413.8 ng/mL
Antinuclear antibody ×160 speckled
Anti-DNA antibody 0.6 IU/mL (<9.0)
Anti-SS-A antibody 89.7 U/ml (<6.0)
MPO-ANCA 1.0 U/mL (<3.4)
PR3-ANCA 1.0 U/mL (<3.4)
Anti-cardiolipin antibody (IgM) <8 U/mL

GFR; glomerular filtration rate, MMP-3; matrix metalloproteinase-3, MPO-ANCA; myeloperoxidase-anti-neutrophil cytoplasmic antibody, PR3-ANCA; proteinase 3-anti-neutrophil cytoplasmic antibody. The values in the parentheses show the normal range.
